# A neurocognitive account of complex PTSD: self-modelling, affective dysregulation, and implications for MDMA-assisted and targeted psychotherapies

**DOI:** 10.1080/20008066.2026.2631358

**Published:** 2026-03-19

**Authors:** Philip Gerrans, Hugh Mcgovern, Jakob Hohwy, Lena K. L. Oestreich

**Affiliations:** a School of Humanities, The University of Adelaide, Adelaide, Australia; b School of Psychology, The University of Queensland, Brisbane, Australia; c IMPACT – The Institute for Mental and Physical Health and Clinical Translation, School of Medicine, Barwon Health, Deakin University, Geelong, VIC, Australia; d Monash Centre for Consciousness and Contemplative Studies, Monash University, Melbourne, VIC, Australia; e Centre for Advanced Imaging, Australian Institute for Bioengineering and Nanotechnology, The University of Queensland, Brisbane, Australia

**Keywords:** Complex post-traumatic stress disorder, self-modelling, C-PTSD, 3,4-methylenedioxymethamphetamine, MDMA-assisted psychotherapy, active inference, Trastorno por estrés postraumático complejo, TEPT-C, psicoterapia asistida con MDMA, inferencia activa, 4-metilendioximetanfetamina, automodelado

## Abstract

**Background**: Complex post-traumatic stress disorder (C-PTSD) is a trauma-related condition characterized by pervasive disturbances in affect regulation, self-concept, and interpersonal functioning that extend beyond the symptom profile of post-traumatic stress disorder (PTSD). While neurobiological studies have implicated limbic, salience, and prefrontal systems in C-PTSD, mechanistic accounts linking these findings to disturbances in self-organization and treatment-relevant processes remain underdeveloped.

**Objective**: This narrative review develops a hypothesis-generating neurocognitive account of C-PTSD grounded in predictive processing and self-modelling frameworks, with particular emphasis on affective dysregulation and disturbances in self-organization.

**Method**: We synthesize clinical, neurobiological, and theoretical evidence to conceptualize PTSD and C-PTSD along a continuum of regulatory stability, and to advance a conceptual model highlighting the role of insula-mediated self-modelling processes. Within this framework, we examine 3,4-methylenedioxymethamphetamine (MDMA)-assisted psychotherapy, as one illustrative intervention that may transiently modulate affective salience, interpersonal trust, and self-referential cognition.

**Results**: The proposed model integrates converging evidence linking insular function to interoception, affective experience, salience processing, and self-referential cognition, and situates disturbances in self-organization as emerging from maladaptive predictive regulation under conditions of prolonged interpersonal adversity. This framework helps reconcile overlapping neurobiological findings across PTSD and C-PTSD while accounting for differences in symptom generalization, relational threat processing, and affective stability.

**Conclusions**: This review delineates a set of testable, mechanistically grounded hypotheses concerning the role of self-modelling processes in C-PTSD. These hypotheses generate specific predictions for future empirical work and inform the design and evaluation of pharmacological and psychotherapeutic interventions that aim to recalibrate affective regulation and self-referential processing in complex trauma presentations.

## HIGHLIGHTS

- Complex post-traumatic stress disorder (C-PTSD) arises from prolonged and/or severe trauma and is marked by difficulties with self-identity, emotional regulation, and interpersonal relationships.
- Our proposed model is based in active inference and highlights the insula, a key brain region for emotional awareness, as central to understanding how C-PTSD disrupts the sense of self.
- We propose that MDMA-assisted therapy may help rebuild a stable sense of self and improve emotional regulation, offering new hope where traditional treatments fall short.

## Abstract (es)

**Antecedentes:** El trastorno por estrés postraumático complejo (TEPT-C) es una afección relacionada con el trauma que se caracteriza por alteraciones generalizadas en la regulación afectiva, el concepto de uno mismo y el funcionamiento interpersonal que van más allá del perfil sintomático del trastorno por estrés postraumático (TEPT). Si bien los estudios neurobiológicos han implicado a los sistemas límbico, de saliencia y prefrontal en el TEPT-C, las explicaciones mecánicistas que relacionan estos hallazgos con las alteraciones en la autoorganización y los procesos relevantes para el tratamiento siguen sin estar suficientemente desarrolladas.

**Objetivo:** Esta revisión narrativa desarrolla una explicación neurocognitiva generadora de hipótesis sobre el TEPT complejo basada en marcos de procesamiento predictivo y automodelado, con especial énfasis en la desregulación afectiva y las alteraciones en la autoorganización.

**Método:** Sintetizamos la evidencia clínica, neurobiológica y teórica para conceptualizar el TEPT y el TEPT complejo a lo largo de un continuo de estabilidad regulatoria, y para promover un modelo conceptual que destaque el papel de los procesos de automodelado mediados por la ínsula. Dentro de este marco, examinamos la psicoterapia asistida con 3,4-metilendioximetanfetamina (MDMA) como una intervención ilustrativa que puede modular de forma transitoria la relevancia afectiva, la confianza interpersonal y la cognición autorreferencial.

**Resultados:** El modelo propuesto integra pruebas convergentes que relacionan la función insular con la interocepción, la experiencia afectiva, el procesamiento de la relevancia y la cognición autorreferencial y sitúa las alteraciones en la autoorganización como resultado de una regulación predictiva desadaptativa en condiciones de adversidad interpersonal prolongada. Este marco ayuda a conciliar los hallazgos neurobiológicos superpuestos entre el TEPT y el TEPT complejo, a la vez que considera las diferencias en la generalización de los síntomas, el procesamiento relacional de las amenazas y la estabilidad afectiva.

**Conclusiones:** Esta revisión delinea un conjunto de hipótesis comprobables y con base mecanicista sobre el papel de los procesos de automodelado en el TEPT complejo. Estas hipótesis generan predicciones específicas para futuros trabajos empíricos y sirven de base para el diseño y la evaluación de intervenciones farmacológicas y psicoterapéuticas que tienen como objetivo recalibrar la regulación afectiva y el procesamiento autorreferencial en casos de trauma complejo.

Complex Post-Traumatic Stress Disorder (C-PTSD) is a trauma-related condition arising from prolonged and/or repeated interpersonal adversity, often occurring during developmentally sensitive periods in childhood or adolescence (Cloitre et al., 2014). In contrast to Post-Traumatic Stress Disorder (PTSD), which is primarily characterized by trauma-specific fear responses and a persistent sense of current threat, C-PTSD includes additional and pervasive disturbances in affect regulation, self-concept, and interpersonal functioning. These features are collectively referred to as disturbances in self-organization (DSO) and are associated with greater functional impairment, higher comorbidity, and poorer treatment outcomes than PTSD alone (Hyland et al., 2017).

The recognition of C-PTSD as a distinct diagnostic entity in the 11th revision of the International Classification of Diseases (ICD-11) reflects growing consensus that existing PTSD models do not fully capture the clinical complexity of trauma arising from sustained interpersonal harm (World Health Organization (WHO), 2019/2021). Individuals with C-PTSD often present with chronic emotional dysregulation, persistent negative self-beliefs, and enduring relational difficulties, including mistrust, avoidance of intimacy, and impaired capacity to use social relationships for affect regulation (Ford & Courtois, 2021; Karatzias et al., 2019). These features pose substantial challenges for treatment engagement and may contribute to reduced efficacy of standard trauma-focused interventions.

Neurobiological studies of PTSD and C-PTSD have consistently implicated limbic, salience, and prefrontal systems involved in emotion regulation, threat detection, and self-referential processing, including the amygdala, medial prefrontal cortex, anterior cingulate cortex, hippocampus, and insular cortex (Hayes et al., 2012; Nicholson et al., 2017; Stopyra et al., 2023). However, interpretation of this literature is complicated by the fact that C-PTSD is defined in part by affective dysregulation and disturbances in self-concept. As a result, overlap between symptom domains and implicated neural systems raises the possibility of circular inference, whereby regions involved in emotion and self-processing are identified precisely because these functions are diagnostically salient. Accordingly, much of the existing neurobiological evidence is correlational and transdiagnostic, and does not, on its own, establish disorder-specific causal mechanisms.

A key challenge for the field is therefore to move beyond descriptive associations and toward integrative frameworks capable of linking clinical phenomenology, neurobiological findings, and treatment-relevant mechanisms without over-interpreting correlational data. Predictive processing and active inference frameworks offer one such approach. These models conceptualize the brain as maintaining hierarchical generative models that guide perception, action, and affect regulation by minimizing prediction errors across multiple timescales (Friston et al., 2017; Parr et al., 2022). Within this framework, the *self* can be understood as a higher-order model that constrains expectations about bodily states, emotions, and social interactions, thereby supporting adaptive regulation and coherent self-experience (Gerrans, 2024; Hohwy, 2016).

From this perspective, disturbances in affect regulation and self-concept characteristic of C-PTSD may reflect disruptions in the development or stability of self-modelling processes, particularly under conditions of prolonged, unpredictable adversity. The insular cortex, given its central role in integrating interoceptive, affective, and salience-related signals, has been proposed as a key neural substrate supporting these processes (Menon & Uddin, 2010; Molnar-Szakacs & Uddin, 2022). Importantly, however, this interpretation remains theoretical and does not imply that observed insular alterations uniquely explain C-PTSD pathology. Despite growing interest in neurobiological correlates of C-PTSD, there remains a lack of integrative models that link clinical phenomenology, brain systems, and treatment-relevant mechanisms within a unified theoretical framework.

The primary aim of this narrative review is to develop a hypothesis-generating neurocognitive account of C-PTSD grounded in predictive processing and self-modelling. Specifically, we seek to synthesize existing clinical and neurobiological evidence to clarify how disturbances in affect regulation, self-concept, and interpersonal functioning may be understood within a self-modelling framework. A secondary aim is to explore how this framework may generate mechanistic hypotheses relevant to interventions that target affect regulation and self-concept. In this context, MDMA-assisted psychotherapy is discussed as one illustrative example of an intervention that may transiently modulate processes central to self-modelling, such as emotional salience, interpersonal trust, and self-referential cognition. Its inclusion is intended to facilitate hypothesis generation rather than to imply clinical endorsement, exclusivity, or superiority over established psychotherapeutic approaches.

We first outline key clinical distinctions between PTSD and C-PTSD and summarize relevant neurobiological findings, with particular attention to affective regulation and self-referential processing networks. We then focus on the insula as a central neural hub implicated across these domains and across trauma-related disorders. Building on this empirical foundation, we subsequently introduce a hypothesis-generating predictive processing and self-modelling account as a theoretical framework for integrating these findings and for interpreting disturbances in self-organization in C-PTSD. Finally, we examine MDMA-assisted psychotherapy as one illustrative intervention through which these hypotheses may be explored, before outlining testable predictions and directions for future research.

## 1. PTSD and C-PTSD: brain–behaviour similarities and differences

PTSD and C-PTSD exhibit considerable overlap in brain activation patterns, functional connectivity, and neural processing during memory, attention, and emotional tasks, yet each condition also presents distinct patterns of neural dysregulation (for a detailed review see Stopyra et al., 2023 and Table 1). Both disorders are characterized by hypervigilance and emotional dysregulation, linked to reduced prefrontal inhibition of the amygdala and brainstem regions, including the periaqueductal grey (Corrigan et al., 2011). C-PTSD often presents with comorbid dissociative symptoms, such as depersonalization and derealization, though these are not core diagnostic features according to ICD-11 criteria. While emotional detachment is part of the disturbances in self-organization symptom cluster, it represents relational difficulties rather than dissociative phenomena *per se*. These symptoms appear to be at least partially driven by excessive top-down prefrontal inhibition of limbic and brainstem regions (Harricharan et al., 2020; Nicholson et al., 2017). Additionally, altered insula activity has been implicated in dissociative symptoms more commonly observed in C-PTSD (Bryant et al., 2021). Depersonalization has been associated with insula dysfunction, particularly involving anterior insula engagement, and with altered prefrontal-insula interactions during emotional processing (Sierra, 2008, 2009; Sierra & David, 2011). In such states, affective experiences may be associated with reduced subjective salience, corresponding to feelings of detachment and diminished self-relevance.

A key limitation of the neurobiological literature reviewed here is the potential for circularity in inference. Because C-PTSD is defined in part by affective dysregulation and disturbances in self-organization, it is unsurprising that implicated neural networks supporting emotion regulation, salience processing, and self-referential cognition, encompass prefrontal, limbic, and paralimbic regions. Such overlap does not imply disorder-specific neural mechanisms and may instead reflect transdiagnostic processes shared across conditions characterized by emotional dysregulation and altered self-experience.

**Figure 1.**
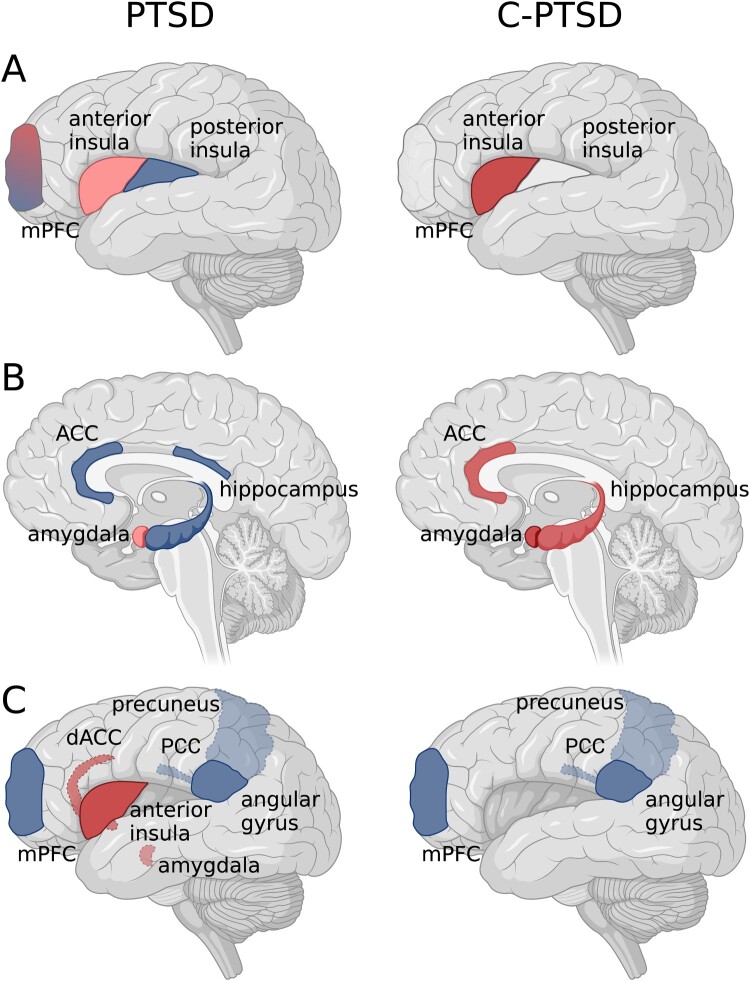
Brain alterations in post-traumatic stress disorder (PTSD) on the left panel and complex-PTSD (C-PTSD) on the right panel. Panel A and B represent findings from task-based functional magnetic resonance imaging (fMRI) and volumetric grey matter studies. Panel C represents functional resting-state fundings. Red = hyperactivation; blue = hypoactivation/ reduced volume, grey = no findings; blue/red = mixed findings. Darker colours represent stronger activation. The model integrates neurobiological and clinical findings summarized in Table 1.

Despite these neural correlates, the causal mechanisms linking brain alterations to clinical symptoms remain unresolved. It is unclear whether regional dysregulation reflects a cause, consequence, or correlate of symptom severity, limiting the interpretability of disorder-specific claims. Accordingly, the evidence reviewed in this section is descriptive rather than explanatory. A comprehensive review of PTSD and C-PTSD brain alterations is beyond the scope of this proposal (Bird et al., 2021), we briefly outline key neural structures implicated in PTSD and C-PTSD (Figure 1 and Table 1) and examine how they may be influenced by MDMA (Table 1). In the following section, we focus on the insula as a candidate integrative hub within affective and self-referential networks, before introducing a broader predictive processing account to interpret these findings.

**Table 1.** Neural alterations reported in PTSD and C-PTSD and hypothesized acute effects of MDMA.

Structure/ network	Post-traumatic stress disorder (PTSD)	Complex post-traumatic stress disorder (C-PTSD)	Processes	Effect of 3,4-methylenedioxymethamphetamine (MDMA)
Amygdala	Increased activation in response to fearful faces (e.g. Bryant et al., 2008; Williams et al., 2006), emotional stimuli and during fear acquisition (Etkin & Wager, 2007) Decreased volume (Morey et al., 2012)	Increased activation in response to fearful faces greater in C-PTSD than in PTSD (Bryant et al., 2021)	Emotional processing	MDMA has been associated with reduced amygdala reactivity while altering its connectivity with the prefrontal cortex (PFC) (Carhart-Harris et al., 2015) and the hippocampus (Carhart-Harris et al., 2015). The hypothesized top-down regulation of fear and stress (Thal & Lommen, 2018), may transiently influence emotional processing of traumatic memories in a calmer and less distressing way.
Hippocampus	Hypoactivation across broad range of memory tasks (Bremner et al., 1999; Hayes et al., 2011) Poorer structural connectivity (Fani et al., 2016) and lower volumes (Logue et al., 2018)	Hyperactivity in the hippocampus, linked to preserved declarative memory, suggests increased sensitivity to emotional stimuli, enhancing memory consolidation (Thomaes et al., 2009)	Memory processing	MDMA has been reported to alter amygdala-hippocampal functional connectivity during acute administration (Carhart-Harris et al., 2015), which influence emotional memory processing in controlled settings.
Insula	Increased activation in response to fearful faces, particular in anterior insula (Bryant et al., 2021) Positive relationship between insular activation and dissociative symptoms (Bryant et al., 2021) Increased resting-state activation in anterior insula (Wang et al., 2016), hypoactivity (Wang et al., 2016) reduced functional connectivity (Zhang et al., 2016) and reduced volume in posterior insula (Clausen et al., 2017)	Increased activation in response to fearful faces, particular in anterior insula; greater in C-PTSD than in PTSD (Bryant et al., 2021), Positive relationship between insular activation and dissociative symptoms (Bryant et al., 2021) Anterior insula hyperactivation in response to affective stimuli (Thomaes et al., 2012)	Self-concept, attentional bias to threat	MDMA has been reported to increase connectivity between the mPFC, posterior cingulate cortex (PCC) and insula (De Gregorio et al., 2021). These changes are hypothesized to transiently enhance feelings of social connectedness and trust, which may facilitate a stronger therapeutic alliance (Carhart-Harris & Goodwin, 2017)
Anterior Cingulate Cortex (ACC)	Hypoactivity in ACC is associated with increased susceptibility to distraction by trauma-related stimuli (Bremner et al., 2004), possibly linked to reduced top-down control reduced volume (Yamasue et al., 2003)	Increased ACC activation during attentional tasks, possibly reflects heightened monitoring and processing of emotional information (Thomaes et al., 2012)	Attentional processing	MDMA has been associated with altered ACC activity during social and affective tasks, which may enhance empathy and social processing (Carhart-Harris et al., 2015)
Medial Prefrontal Cortex (mPFC)	Increased (Bryant et al., 2008) and decreased (Shin et al., 2005; Williams et al., 2006) activation in response to fearful faces		Emotional processing	MDMA has been reported to increases connectivity between the mPFC, PCC and insula (De Gregorio et al., 2021). These effects may transiently modulate self-referential and social cognitive processing (Carhart-Harris & Goodwin, 2017)
Default Mode Network (DMN)	Reduced activity, particularly the mPFC, disrupts self-referential processes and emotional regulation & contributes to rigid, trauma-focused thought patterns (Burback et al., 2024; Daniels et al., 2011)	Decreased functional connectivity between the PCC and the mid-cingulate cortex (MCC), as well as between the PCC and putamen, compared to both PTSD and controls (Kim et al., 2024)	Self-referential thinking, introspection, social cognition	MDMA has been associated with altered DMN activity during acute administration, disrupting maladaptive self-referential loops and fostering a mental state that promotes reorganization of thought patterns, allowing engagement with trauma in adaptive ways during therapy (Carhart-Harris & Goodwin, 2017)
Salience Network (SN)	Dysregulation in SN causes hypervigilance, heightened threat detection, and difficulty disengaging from trauma cues (Akiki et al., 2017) Disrupted connectivity between the SN, DMN, and central executive network worsens maladaptive self-referential processing and emotional regulation	N/A	Detecting and prioritizing important internal and external stimuli	MDMA has been shown to alter SN connectivity. These effects may transiently influence threat detection and salience attribution, which is essential for processing trauma effectively (Walpola et al., 2017)

Note: Reported MDMA-related effects reflect acute or short-term neural changes observed in PTSD or healthy samples. These associations should not be interpreted as evidence of therapeutic efficacy, disorder-specific mechanisms, or superiority over other emotion-regulation or self-focused interventions in C-PTSD.

## 2. Insula, self-modelling, and trauma

Having outlined convergent brain–behaviour findings and their interpretive limits, we now introduce a self-modelling account to integrate these observations, focusing on the insula as a candidate neural hub for disturbances in affective regulation and self-experience. The concept of self-modelling helps explain the insula’s pervasive yet elusive role not only in PTSD and C-PTSD but also in other conditions characterized by disturbances in affective experience and self-awareness, such as borderline personality disorder, which shares overlapping features related to affective instability and disturbances in self-experience. Notably, the sense of being the subject of experience is typically a subtle and evanescent background phenomenon, reflecting the self’s stability as both the source and target of cognition. For this reason, self-awareness usually functions as a ‘subtle coefficient’ of experience (Billon, 2023). However, when self-modelling circuitry becomes dysregulated, marked by unpredictable and intractable states, it disrupts this background process, demanding attentional and executive resources and forcing subjectivity into the foreground of cognition.

In brief, we propose that the difference between PTSD and C-PTSD reflects variations in the stability and effectiveness of self-modelling. In PTSD, trauma occurs when the individual has already developed a stable self-model. In neural network terms, the insula-modulated neural networks have been trained to predict characteristic adaptive state transitions. Translated into the framework of ‘active inference’ the brain stabilizes a generative model that predicts the bodily and affective consequences of regulatory action (Corcoran et al., 2020). However, in traumatic episodes, persistent mismatches between the sensory information predicted by the self-model’s priors and the actual sensory consequences of action can disrupt this process, making it impossible to establish a predictable mapping of experience, ultimately rendering the event traumatic. This model acknowledges that it remains an open empirical question whether self-model fragility precedes trauma, is caused by it, or arises epiphenomenally from other psychopathological mechanisms. For instance, a child abused by a trusted adult has no prior framework to interpret or manage such an experience. As a result, they become trapped in escalating negative affect, unable to predict, regulate, or integrate the experience into their self-model. This dysregulation leads to maladaptive regulatory strategies, one of which is dissociation.

Dissociation is a multifaceted phenomenon conceptualized differently across theoretical frameworks, including structural dissociation theory, which posits trauma-induced division of the personality (van der Hart et al., 2006); betrayal trauma theory, which emphasizes dissociation as adaptive unawareness of abuse by caregivers (Freyd, 1996); and neurobiological models focusing on altered brain function during threat (Sierra & David, 2011). For the purposes of our self-modeling framework, we draw on a neurobiological account proposing that dissociation involves an involuntary inhibitory response that deactivates the anterior insula, preventing sensory information from being integrated into the self-model (Medford et al., 2016). This account may be particularly applicable to depersonalization and derealization symptoms rather than all dissociative phenomena. In C-PTSD, which often arises from prolonged, unpredictable, and dysregulating adversity, a stable self-model fails to develop. However, this does not exclude the possibility that abuse occurring in adulthood could disrupt and potentially dismantle an already stable self-model. The key issue is not merely that the world is unpredictably adverse (i.e. traumatic) but that when adversity is sustained, the development of a stable self-model with adequate coping mechanisms is impaired. As a result, C-PTSD and related conditions are often marked by chronic dysregulation and maladaptation across various contexts, making it difficult for individuals to accurately identify, appraise, and respond to self-relevant experiences. Supporting this, individuals with C-PTSD often struggle to implement and sustain effective affect regulation over extended time scales (Hyland et al., 2017). Recent neuroimaging evidence also supports insula involvement in dissociation: Harricharan et al. (2020) found altered anterior insula connectivity in the dissociative subtype of PTSD, and Nicholson et al. (2017) demonstrated that individuals with dissociative PTSD show distinct insula activation patterns compared to those with non-dissociative PTSD during threat processing.

## 3. A self-modelling account of PTSD and C-PTSD: a continuum of regulatory stability

**Figure 2.**
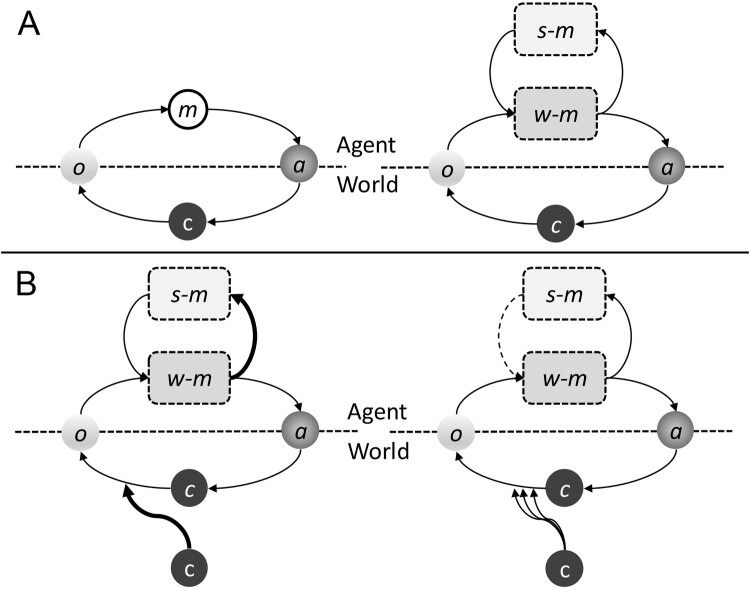
Hypothesis-generating conceptual model illustrating differences in self-modelling stability and affect regulation in PTSD and C-PTSD. (A) The model depicts how agents develop and update their self-model (*s-m*) and world-model (*w-m*). (B) In PTSD (left), the self-model remains relatively intact but is highly volatile, leading to strong but context-specific fear responses. In C-PTSD (right), self-modelling is disrupted, resulting in unstable identity, maladaptive regulation, and persistent interpersonal difficulties; *o* = observation; *m* = model; *a* = action; c = constraint.

Taken together, the evidence reviewed above indicates substantial overlap in the neural systems supporting affect regulation, salience processing, and self-referential cognition in PTSD and C-PTSD, alongside differences in the stability and generalization of these processes. However, these findings remain largely descriptive and do not, on their own, establish causal mechanisms. To integrate these observations, we propose a hypothesis-generating self-modelling account that situates PTSD and C-PTSD along a continuum of regulatory stability. This conceptual framework is illustrated in Figure 2, which schematically depicts this model across increasing levels of regulatory instability. In all panels, the agent is separated from the world by a dashed boundary. Sensory observations (o) are generated by the world and sampled by the agent, while actions (a) influence the environment in ways intended to minimize affective and cognitive uncertainty. Within the agent, internal models support prediction and regulation: the world-model (*w*-*m*) encodes expectations about environmental states and their consequences, while the self-model (*s*-*m*) encodes expectations about the agent’s own regulatory capacities, affective responses, and action-outcome contingencies. The circular perception-action loop reflects ongoing active inference, whereby predictions guide action and sensory feedback updates internal models. Constraints (c) represent limiting factors on regulatory capacity, including environmental, physiological, developmental, and social factors. These constraints shape the confidence with which regulatory strategies can be selected and sustained over time.

Figure 2 illustrates this framework across differing levels of self-modelling stability. In Figure 2A, the agent’s self-model and world-model are relatively stable and mutually informative, allowing affective regulation to generalize across contexts. In PTSD (Figure 2(B), left), the self-model remains largely intact but becomes volatile and context-sensitive, resulting in strong, context-specific threat responses despite otherwise preserved regulatory capacity. By contrast, in C-PTSD (right), self-modelling is more globally disrupted. Here, multiple interacting constraints limit the reliability of regulatory strategies, contributing to unstable self-representation, maladaptive affect regulation, and persistent interpersonal difficulties. The additional constraints depicted in the C-PTSD panel reflect the cumulative impact of limiting contextual factors rather than discrete traumatic events. This framing provides a conceptual basis for understanding PTSD and C-PTSD as differing primarily in the stability and generalizability of self-modelling processes.

Within this framework, differences between PTSD and C-PTSD can be understood primarily through the lens of affective regulatory stability. Effective emotional regulation requires confidence and competence in managing emotionally salient situations, capacities that develop through a history of successful encounters that reinforce predictable social engagement strategies and shape a stable self-model (Gross, 2015). In active inference terms, a stable self-model lowers the perceived risk associated with regulatory strategies, fostering resilient affective responses. Because the model predicts adequate coping with adversity the subject is able to (i) tolerate transient episodes (ii) maintain the capacity for exploratory response. By contrast, C-PTSD is proposed to arise from prolonged, inconsistent adversity that prevents the consolidation of effective regulatory patterns. A child growing up in such an environment learns that emotional states can shift unpredictably and uncontrollably, with no reliable strategy to mitigate distress. In extreme cases, dissociation or capitulation may become the only available coping mechanisms. Consequently, the child fails to develop a stable self-model capable of predicting and executing successful coping strategies, resulting in a fragile and negative self-concept.

Since the *self* is inferred to be the source and target of regulation, a chronically dysregulated internal and external environment undermines confidence in one's independent viability. Under typical developmental conditions, this would be ameliorated by reliance on trusted others for affective regulation, particularly during infancy and adolescence, critical periods for shaping adaptive emotional responses (Fonagy & Luyten, 2009). However, in C-PTSD, inconsistent, insecure, or adverse relationships disrupt this developmental trajectory, leading to (i) an unstable and maladaptive self-model, incapable of sustaining long-term regulatory strategies, (ii) over-reliance on external sources of regulation, resulting in intense, labile, and insecure attachments that further destabilize affective regulation. These disruptions are reflected clinically in the ICD-11 construct of disturbances in self-organization (Hyland et al., 2017).

## 4. Targeting self-organization in current practice

The neurocognitive disturbances described above, particularly the fragile self-model and affective dysregulation, are not uniquely targeted by pharmacological interventions. Established psychotherapeutic modalities have long recognized the need to address disturbances in self-organization symptoms prior to, or alongside, trauma memory processing.

Skills Training in Affective and Interpersonal Regulation (STAIR) combined with narrative therapy has demonstrated efficacy for individuals with childhood abuse-related PTSD, with effects maintained at follow-up (Cloitre et al., 2010; Karatzias et al., 2023; Niwa et al., 2022). This phase-based approach first builds emotional regulation and interpersonal skills before processing traumatic memories. The initial phase explicitly teaches skills for identifying and managing emotions (targeting affect dysregulation), while also addressing interpersonal patterns through modules on assertiveness, flexibility in relationships, and managing interpersonal conflict (targeting disturbances in relationships). By establishing these regulatory capacities before trauma processing, STAIR directly targets the disturbances in self-organization characteristic of C-PTSD.

Dialectical Behavior Therapy (DBT), originally developed for borderline personality disorder, has been adapted for PTSD (DBT-PTSD) and shows efficacy for addressing emotion dysregulation in complex trauma populations (Bohus et al., 2020). DBT combines cognitive–behavioural techniques with mindfulness practices, teaching four core skill sets: mindfulness, distress tolerance, emotion regulation, and interpersonal effectiveness. These directly map onto C-PTSD symptom domains: emotion regulation skills target affect dysregulation; interpersonal effectiveness addresses relationship disturbances; and mindfulness cultivates present-moment awareness and self-observation, potentially counteracting dissociative tendencies and supporting more coherent self-experience.

Schema Therapy targets early maladaptive schemas i.e. deeply held negative beliefs about self and others that develop in childhood (e.g. ‘I am defective,’ ‘I am unlovable,’ ‘Others will abandon me’). These schemas directly correspond to the negative self-concept domain of C-PTSD. Schema Therapy uses cognitive, experiential, and relational techniques to identify, challenge, and modify these core beliefs, while also addressing the maladaptive coping modes that maintain them. Preliminary evidence supports its application (Ubico et al., 2025) to complex trauma presentations (Cockram et al., 2010; Lian et al., 2024).

Compassion-Focused Therapy (CFT) specifically addresses shame and self-criticism, which are central to the negative self-concept seen in C-PTSD (Lee & James, 2012). CFT proposes that individuals with trauma histories often have underdeveloped self-soothing capacities and overactive threat systems. Through compassionate mind training, imagery exercises, and cultivation of self-compassion, CFT aims to activate the affiliative/soothing system, directly targeting the harsh self-perception and inability to self-reassure characteristic of C-PTSD. This approach may be particularly relevant for individuals whose negative self-concept is maintained by chronic self-criticism and shame.

Taken together, these approaches demonstrate that disturbances in self-organization are already implicitly targeted in current practice, providing an important context for evaluating whether novel interventions engage similar mechanisms through different pathways. We do not suggest that MDMA-assisted therapy should be considered to the exclusion of these approaches; rather, we propose it as a potentially valuable addition to the treatment armamentarium that merits further investigation alongside these established interventions. The comparative efficacy of these different approaches for C-PTSD remains an open empirical question requiring direct investigation.

## 5. MDMA-Assisted psychotherapy as an illustrative example

The preceding neurobiological review does not warrant direct therapeutic inference. Rather, it motivates the development of mechanistic hypotheses concerning interventions that may transiently modify affective regulation, salience processing, and self-referential cognition. In the following section, we examine MDMA-assisted psychotherapy as one illustrative case through which such hypotheses may be explored, without implying exclusivity, clinical endorsement, or superiority over other interventions targeting similar processes.

MDMA has shown promising results in the treatment of PTSD, with a complex safety profile that includes both therapeutic benefits and recognized risks (Henner et al., 2022). Clinical trials have demonstrated significant improvements in patients with moderate to severe PTSD through MDMA-assisted psychotherapy (Mitchell et al. 2023a; Jerome et al., 2020). It is also noteworthy that participants enrolled in Phase 3 MDMA-assisted psychotherapy trials for PTSD frequently reported histories of childhood trauma and multiple traumatic events (Jerome et al., 2020; Mitchell et al., 2023b). Although these studies did not assess C-PTSD as a diagnostic category, it is likely that a substantial proportion of participants would have met ICD-11 criteria for C-PTSD. This observation does not permit conclusions regarding the efficacy of MDMA-assisted therapy for C-PTSD *per se*, but it underscores the relevance of examining whether mechanisms of change observed in PTSD trials may extend to trauma presentations characterized by disturbances in self-organization.

MDMA, a synthetic stimulant often classified as an empathogen-entactogen, shares some properties with classical psychedelics (Nichols, 1986). Like psilocybin and LSD, MDMA acts on 5-HT_2A_ receptors (Capela et al., 2007). However, while the psychoactive effects of classical psychedelics are primarily mediated by 5-HT_2A_ receptor activation, MDMA differs by additionally enhancing the release and reuptake of dopamine, norepinephrine (Verrico et al., 2007), and oxytocin (Kirkpatrick et al., 2014). MDMA predominantly releases serotonin through 5-HT_1B_ receptor agonism, a mechanism thought to enhance social engagement and trust (Rempel et al., 1993).

Due to its distinct but overlapping mechanisms of action, MDMA produces a subjective experience similar to, though milder than, classical psychedelics, characterized by increased empathy (Hysek et al., 2014), pro-sociality (Borissova et al., 2021), and perceptual alterations (Vollenweider et al., 2002). Research suggests that MDMA enhances fear-extinction learning and promotes acceptance and conflict tolerance (Young et al., 2015). These effects are driven by its influence on monoamine systems, particularly serotonin release via presynaptic transporters, leading to significant increases in serotonin and dopamine (Schenk & Highgate, 2021). As a result, MDMA has become a key focus in discussions on PTSD treatment, particularly for symptom reduction and recovery (Smith et al., 2022).

Despite growing evidence supporting MDMA's effectiveness in treating PTSD, including treatment-resistant cases (Colcott et al., 2024), there remains a critical gap in research on MDMA-assisted therapy for C-PTSD. The literature to date has largely focused on PTSD populations, with few studies clarifying whether mechanisms of change in MDMA-assisted therapy generalize to the distinct relational and self-concept disturbances that characterize C-PTSD. Given these differences, it remains unclear whether observed therapeutic effects reflect trauma-specific mechanisms, broader transdiagnostic processes, or context-dependent interactions between pharmacology and psychotherapy. Nevertheless, several lines of evidence suggest that MDMA may transiently modulate processes central to affect regulation, self-referential cognition, and interpersonal engagement. In healthy volunteers, MDMA has been shown to enhance feelings of authenticity and facilitate autobiographical disclosure (Baggott et al., 2016), effects that may be relevant to individuals with C-PTSD who experience fragmentation of self-experience and impaired interpersonal trust. At the neurobiological level, MDMA has been associated with altered salience-network organization, including reduced insular integration and changes in bodily and affective experience (Walpola et al., 2017). These findings suggest that MDMA may temporarily disrupt maladaptive patterns of salience attribution and self-referential processing, potentially creating windows for therapeutic reorganization of self-models within a supportive psychotherapeutic context. Importantly, such effects do not imply therapeutic specificity for C-PTSD and should be interpreted as hypothesis-generating rather than explanatory. On this basis, we next outline a preliminary neurocognitive framework to explore how MDMA-assisted psychotherapy might interact with the pathophysiology of C-PTSD.

## 6. An integrated model of MDMA therapy for C-PTSD

Thus far, we have examined brain alterations in PTSD and C-PTSD relative to healthy controls and outlined acute and post-acute neurobiological changes brought about by MDMA. Synthesizing these findings, both PTSD and C-PTSD exhibit dysregulated emotional processing, maladaptive self-referential cognition (e.g. dissociation, rigid negative self-concept), aberrant salience processing, and impaired social cognition, though the degree and nature of these deficits vary between the two disorders. Experimental and clinical studies indicate that acute MDMA administration modulates emotional threat processing and social-affective cognition, including reduced amygdala responsivity to negative emotional stimuli, enhanced affiliative responding, increased feelings of authenticity, and greater interpersonal openness (Baggott et al., 2016; Borissova et al., 2021; Dumont et al., 2009; Hysek et al., 2014; Mitchell et al. 2023a). We hypothesize that, within a structured psychotherapeutic context, these acute effects may temporarily loosen rigid self-referential priors and salience assignments, thereby reducing reliance on avoidance-based or dissociative coping strategies and facilitating corrective learning during therapy. These interpretations are consistent with MDMA’s documented effects on emotional processing, prosocial engagement, and experience-dependent plasticity (Carhart-Harris et al., 2018; Hysek et al., 2014) and may therefore be mechanistically relevant to clinical presentations such as C-PTSD, in which disturbances of self-coherence and interpersonal trust are prominent.

Importantly, potential therapeutic effects of MDMA appear to depend not only on neurochemical changes, but on the psychotherapeutic context in which MDMA is administered, consistent with longstanding evidence on the interaction between pharmacology, expectancy, and therapeutic setting (‘set and setting’) in psychedelic-assisted interventions (Hartogsohn, 2017). Within the present self-modelling framework, the self-model is proposed as a unifying mechanism coordinating emotional, cognitive, and social information across typical and pathological cognition. Failure of this integrative process in C-PTSD may contribute to symptom persistence and treatment resistance.

Neurobiologically, converging evidence implicates the posterior insula in basic interoceptive processing and the anterior insula in affective experience and affectively inflected self-awareness (Gerrans, 2024; Kuehn et al., 2016; Lamm & Singer, 2010; Namkung et al., 2017; Simmons et al., 2013; Zhao et al., 2023). This functional gradient supports the interpretation of the insula as a central hub for self-modelling, integrating bodily, emotional, and contextual information to guide adaptive behaviour (Betti & Aglioti, 2016). Taken together, these observations suggest that MDMA-assisted psychotherapy may transiently modify maladaptive neural priors related to affective salience and self-referential processing, potentially supporting reorganization of self-models within a secure therapeutic relationship. This account remains to be tested empirically. For example, longitudinal neuroimaging studies could assess whether MDMA-assisted psychotherapy is associated with changes in insula connectivity during affective and self-referential tasks, and whether such changes track improvements in emotion regulation and self-concept.

## 7. Clinical implications and future directions

Our proposed self-modelling framework yields several testable hypotheses concerning the mechanisms through which MDMA-assisted psychotherapy may influence symptoms of C-PTSD. These hypotheses are intended to guide empirical investigation rather than immediate clinical application. Central to the model is our first hypothesis that MDMA administration transiently alters affective and salience-processing systems, including reduced amygdala responsivity to threat-related stimuli and altered integration of the anterior insula within salience and social cognition networks (Dumont et al., 2009; Hysek et al., 2014; Walpola et al., 2017). These acute neural effects are expected to correlate with short-term reductions in hypervigilance, affective reactivity, and dissociative responding during therapeutic engagement, consistent with findings from neuroimaging and experimental studies of MDMA in humans (Mitchell et al. 2023b)

A second hypothesis concerns self-referential cognition. C-PTSD is characterized by rigid, negatively valanced self-models and pervasive expectations of interpersonal threat (Cloitre et al., 2014; Hyland et al., 2017). We propose that MDMA-assisted psychotherapy may temporarily reduce the precision of these maladaptive self-referential priors, thereby loosening avoidance-based or dissociative coping strategies during therapy sessions. This hypothesis is grounded in evidence that MDMA enhances autobiographical disclosure, perceived authenticity, and emotional openness in both healthy participants and clinical samples (Baggott et al., 2016; Borissova et al., 2021). Behaviourally, such effects may manifest as increased engagement with emotionally salient material and reduced experiential avoidance, while clinically they may be reflected in short-term changes in affect regulation and self-concept coherence. These changes are expected to be transient in the absence of structured psychotherapeutic integration.

A further implication of the model is that therapeutic alliance plays a mechanistic role, rather than merely a supportive one, in treatment response. Therapeutic alliance is a robust transdiagnostic predictor of psychotherapy outcomes, but is often compromised in individuals with C-PTSD due to interpersonal mistrust and heightened threat sensitivity (Ehlers et al., 2004; Popolo et al., 2025). We therefore hypothesize that MDMA-facilitated reductions in threat perception may enhance the formation of therapeutic alliance, which in turn mediates improvements in disturbances in self-organization. This mediation pathway is consistent with evidence that MDMA increases interpersonal trust, empathy, and affiliative responding (Hysek et al., 2014) and aligns with relational models of trauma recovery emphasizing interpersonal safety as a prerequisite for affect regulation and self-integration (Fonagy & Luyten, 2009).

The model further generates predictions regarding the generalization and durability of treatment effects. Acute increases in perceived safety, trust, and emotional openness following MDMA administration are unlikely to generalize automatically beyond the therapeutic context. Instead, sustained improvement is hypothesized to depend on integration processes that explicitly target interpersonal expectations and self-referential beliefs, enabling newly acquired experiences of safety and agency to update longer-term self-model priors. This prediction is consistent with learning-based accounts of trauma recovery and memory reconsolidation, which emphasize the necessity of repeated, contextually embedded corrective experiences for durable change (Ehlers et al., 2004; Feduccia & Mithoefer, 2018; Traynor et al., 2022).

Finally, the framework predicts mechanistic differences between PTSD and C-PTSD. Whereas symptom improvement in PTSD may be driven primarily by reductions in trauma-specific fear responses and enhanced extinction learning, improvement in C-PTSD is hypothesized to depend more strongly on changes in self-concept coherence, affect regulation, and interpersonal functioning (Cloitre et al., 2014; Karatzias et al., 2019). These differential pathways can be empirically tested using longitudinal designs that combine behavioural assessment, validated measures of disturbances in self-organization, and neurobiological indices of threat, salience, and social cognition.

Taken together, this framework delineates a set of falsifiable predictions linking affective neurobiology, self-modelling, and psychotherapeutic context. While MDMA-assisted psychotherapy is used here as an illustrative case, the mechanisms outlined are not specific to MDMA and may be engaged by other interventions targeting affect regulation and self-concept through different pathways. Future research should focus on directly testing these predictions and clarifying the conditions under which transient neurobiological changes translate into enduring clinical benefit.
